# Empowering Naringin’s Anti-Inflammatory Effects through Nanoencapsulation

**DOI:** 10.3390/ijms25084152

**Published:** 2024-04-09

**Authors:** Andreia Marinho, Catarina Leal Seabra, Sofia A. C. Lima, Alexandre Lobo-da-Cunha, Salette Reis, Cláudia Nunes

**Affiliations:** 1 LAQV, REQUIMTE, Faculdade de Farmácia, Universidade do Porto, R. Jorge de Viterbo Ferreira 228, 4050-313 Porto, Portugal; aierdnamarinho@gmail.com (A.M.); cati.seab@gmail.com (C.L.S.); shreis@ff.up.pt (S.R.); 2 LAQV, REQUIMTE, Faculdade de Ciências, Universidade do Porto, R. do Campo Alegre s/n, 4169-007 Porto, Portugal; 3 LAQV, REQUIMTE, Instituto de Ciências Biomédicas Abel Salazar, Universidade do Porto, R. Jorge de Viterbo Ferreira 228, 4050-313 Porto, Portugal; slima@icbas.up.pt; 4 Departamento de Microscopia, Instituto de Ciências Biomédicas Abel Salazar, Universidade do Porto, R. Jorge de Viterbo Ferreira 228, 4050-313 Porto, Portugal; alcunha@icbas.up.pt

**Keywords:** anti-inflammatory activity, naringin, lipid nanoparticles, macrophages, hyaluronic acid

## Abstract

Abundant in citrus fruits, naringin (NAR) is a flavonoid that has a wide spectrum of beneficial health effects, including its anti-inflammatory activity. However, its use in the clinic is limited due to extensive phase I and II first-pass metabolism, which limits its bioavailability. Thus, lipid nanoparticles (LNPs) were used to protect and concentrate NAR in inflamed issues, to enhance its anti-inflammatory effects. To target LNPs to the CD44 receptor, overexpressed in activated macrophages, functionalization with hyaluronic acid (HA) was performed. The formulation with NAR and HA on the surface (NAR@NPs_HA_) has a size below 200 nm, a polydispersity around 0.245, a loading capacity of nearly 10%, and a zeta potential of about 10 mV. In vitro studies show the controlled release of NAR along the gastrointestinal tract, high cytocompatibility (L929 and THP-1 cell lines), and low hemolytic activity. It was also shown that the developed LNPs can regulate inflammatory mediators. In fact, NAR@NPs_HA_ were able to decrease TNF-α and CCL-3 markers expression by 80 and 90% and manage to inhibit the effects of LPS by around 66% for IL-1β and around 45% for IL-6. Overall, the developed LNPs may represent an efficient drug delivery system with an enhanced anti-inflammatory effect.

## 1. Introduction

Responsible for the bitter taste of some citrus fruits, [1] naringin (4′,5,7-trihydroxyflavanone-7-rhamnoglucoside—NAR) is the main flavonoid found in grapefruit [1,2,3,4]. It is a multifunctional compound [5] that, similarly to other bioactive compounds present in fruits and vegetables, has attracted the interest of the scientific community due to the wide range of beneficial effects on health [2,4]. These beneficial effects include antioxidant [1,2,4,6,7,8], anti-inflammatory [2,4,5,7,9], anticancer [1,6,8,10], antidiabetic [9,10,11], antiatherosclerotic [10,11], renoprotective [11], cardioprotective [9,10,11], hepatoprotective [10], neuroprotective, and [9,10] antiulcer activities [4,6], and lipid-lowering in the blood [1,8,10], among others. Additionally, it has also been shown that NAR can protect against joint destruction in an osteoarthritis model [5]. Through in vitro studies, carried out with primary murine chondrocytes, it is evident that treatment with NAR leads to a reduction in the levels of inflammatory and catabolic markers. Furthermore, in vivo (osteoarthritis in a surgically induced mice model) results showed that NAR treatment was able to decrease the degradation of the cartilage matrix and protect against the development of osteoarthritis. NAR was even able to attenuate nuclear factor kappa B levels, both in vitro and in vivo [5]. Other studies show the potential application of naringin in the treatment of inflammation. The review by Yu et al., for example, reports the potential application of NAR in the treatment of various pathologies, such as osteoporosis [3].

The oral administration of drugs is still the most accepted form due to its advantages, such as the fact that it is painless and allows easy self-administration [12]. However, despite all previously reported beneficial effects, the therapeutic role of flavonoids still depends on an improvement in the pharmacokinetic profile of this class of compounds after oral administration [13]. The gastrointestinal tract presents chemical, enzymatic, and cellular barriers that limit the effectiveness of orally administered drugs [12]. In the particular case of NAR, the literature reports a low oral bioavailability [1], which is due to the extensive phase I and phase II metabolism in the liver [9,10], and its decomposition by the intestinal microflora [9,10,13,14,15].

To overcome these limitations, alternative formulations of NAR to protect it from degradation in the gastrointestinal tract, thus improving its bioavailability, are needed [16]. A promising technology for bioactive compound delivery is nanoparticles [17], which have the potential to improve the stability and solubility of encapsulated molecules [17,18], promote transport across membranes, and prolong circulation times [18], while still being able to allow the delivery to target sites [19].

The literature reports some works where NAR-delivery nanosystems were developed. Wang et al. described that solid lipid nanoparticles loaded with NAR have a greater hepatoprotective effect than free NAR, against aflatoxin B1-induced hepatocellular carcinoma [20]. Imam et al. showed that hybrid nanoparticles soya-lecithin and chitosan loaded with NAR increase the antimicrobial activity against *Escherichia coli*, compared to free NAR [21]. If we focus only on the anti-inflammatory activity of NAR, we can also find some examples reported in the literature. The work developed by Pleguexuelos-Villa et al. shows that ultra-deformable liposomes with NAR are more effective in the treatment of skin inflammation than free NAR or betamethasone cream [22]. Mohanty et al. showed that polymeric nanoparticles loaded with NAR can lower the arthritic score in treated rats more efficiently than free NAR [16].

The strategy presented in this work is based on the development of nanostructured lipid carriers (NLC) for the delivery of NAR with a focus on treating inflammation. One of the great advantages of this type of transporter is that they can be modified on the surface so that they can be directed to target sites, in this case, activated macrophages. Functionalization with hyaluronic acid (HA) was considered since activated macrophages overexpress the CD44 receptor for which hyaluronic acid has high binding affinity. The results obtained show that the use of nanoparticles (NPs) for NAR administration is an advantage since a controlled release along the gastrointestinal tract is achieved and using an inflammation model (THP-1 cell line) it has been shown that NAR@NPs can regulate inflammatory mediators.

## 2. Results and Discussion

### 2.1. Nanoparticles Characterization

Table 1 shows the results obtained for the physicochemical characterization of the developed nanoparticles, in terms of size, polydispersity index, zeta potential, and encapsulation efficiency. The introduction of CTAB into the matrix results in a reduction in the size of the LNPs (about 150 nm). CTAB is a surfactant and thus can lead to a rearrangement in the matrix of LNPs, which is reflected in smaller sizes than LNPs without CTAB. Furthermore, the introduction of the functionalization with HA does not cause significant variations in the size of the nanoparticles, compared to LNPs with CTAB. For the three different types of LNPs, the presence of NAR in the matrix does not induce changes in their sizes.

**Figure 1:**
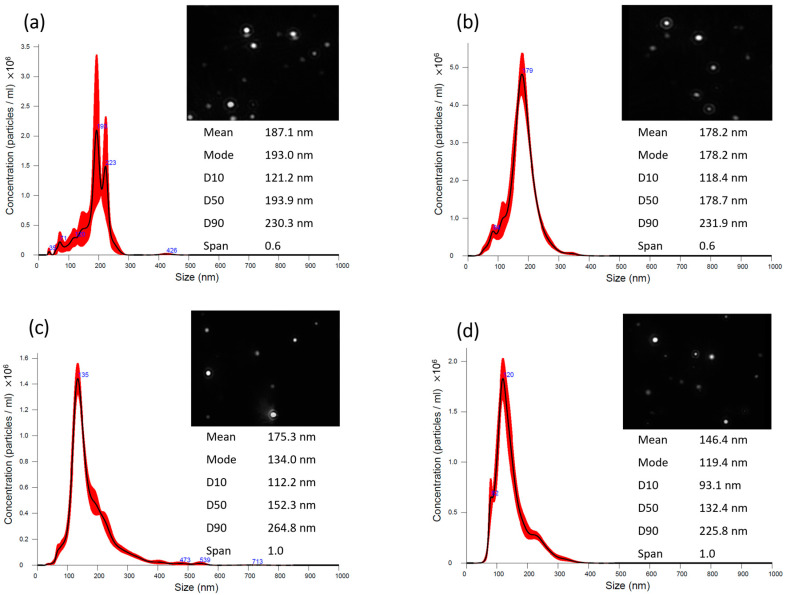
Particle size determination using NTA: (**a**) NPs; (**b**) NAR@NPs, (**c**) NPs_HA_, and (**d**) NAR@NPs_HA_. Average size distribution following 5 measurements. The images correspond to NTA video frames.

Given the difference obtained between the three types of LNPs, the nanoparticle tracking analysis (NTA) technique (Figure 1) was used to evaluate in greater detail the results obtained by the DLS technique. With this technique, the results obtained for NPs and NPs_HA_ (without and with NAR) indicate an average size between 140 and 190 nm, which are much lower than the values obtained using DLS. Larger particles contribute to greater light scattering, which can justify the results obtained by DLS, even if particles with larger sizes are fewer in number, the final value of the reading will be affected and, thereby, deviated to larger average sizes [23]. NTA tracks particle by particle through image analysis and provides simultaneous, multiparameter analysis of nanoparticles in liquid suspensions, concerning size distribution and direct and real-time visualization.

The PDI obtained for the formulations was around 0.2, which indicates that the LNP sizes are uniform, despite the self-assembly ultrasound method [24,25]. It is again verified that the introduction of HA functionalization does not cause significant variations in this parameter. In addition to the PDI, the values of the percentiles (D10, D50, and D90) obtained by the NTA technique can be used to calculate the span value, i.e., (D90 − D10)/D50, which relates the distribution of each sample with the median [26]. The values obtained for NPs and NAR@NPs was 0.6 and for nanoparticles functionalized with HA, NPs_HA_, and NAR@NPs_HA_ it was 1. The literature reports that span values smaller than 1 indicate that the sample is monodisperse [27]. Taken together, the PDI and span results indicate that the developed formulations are monodisperse.

The stability of LNPs in suspension depends on the zeta potential, [27] a standard analytical measure that, in addition to stability, can provide information on the circulation time of LNPs, biocompatibility, and cell permeability, among others [28,29]. The reference value for the stability of formulations is 30 mV, in absolute value [30]. Table 1 shows that the zeta potential obtained for NPs and NAR@NPs is close to the reference value so that the nanoformulations are considered electrostatically stable. As expected, the introduction of CTAB into the lipid matrix shifted the zeta potential value in the opposite direction, yet it remained very close to the reference value, showing, once again, that the nanoformulations are electrostatically stable. On the other hand, the introduction of functionalization with HA leads to a reduction in zeta potential values to values close to neutrality. Since HA has a negative charge [31,32], it binds to the positive surface of LNPs through electrostatic interaction. The results obtained for LNPs functionalized with HA are in line with other examples reported in the literature [33].

The encapsulation efficiency obtained, 30–40%, is due to the poor solubility of NAR both in water and lipid phases, which leads to low rates of encapsulation [34,35,36]. Nevertheless, the loaded amount is protected from extensive metabolism and decomposition in the body, in opposition to what happens with free NAR [9,10,13,14,15].

**Figure 2:**
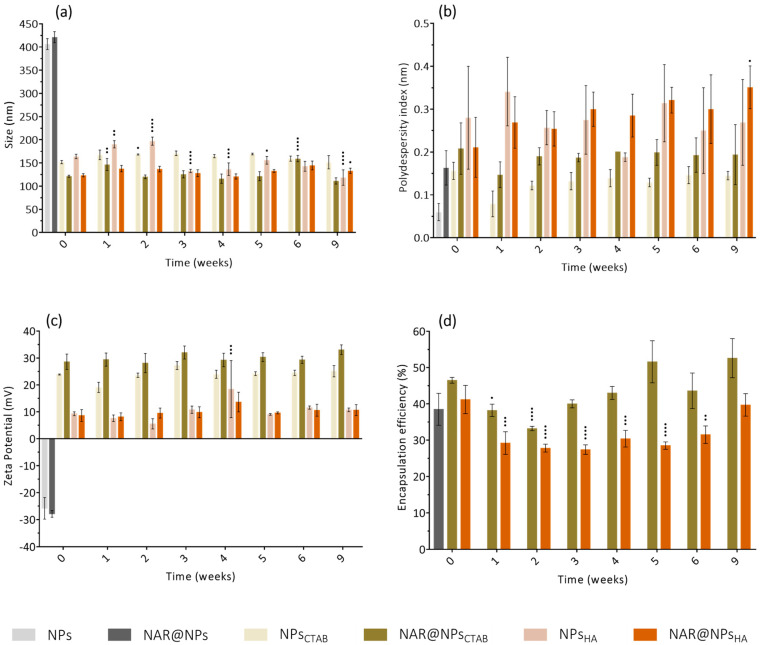
Physical stability of developed NPs over time (0, 1, 2, 3, 4, 5, 6, and 9 weeks): (**a**) size, (**b**) polydispersity index, (**c**) zeta potential, and (**d**) encapsulation efficiency. Values represent mean ± SD (*n* = 3). Differences between groups were assessed using two-way ANOVA followed by Dunnett test. ^•^ *p* < 0.05, ^••^ *p* < 0.01, ^•••^ *p* < 0.001 and ^••••^ *p* < 0.0001 relatively to the correspondent week 0.

Figure 2a presents the results obtained for the stability studies of the developed LNPs over time (0, 1, 2, 3, 4, 5, 6, and 9 weeks). For NPs and NAR@NPs, there is a loss of stability after a few days, with a visual difference in the viscosity of the nanoformulations. NPs_CTAB_ and NAR@NPs_CTAB_ are stable over 9 weeks. The PDI values (Figure 2b) corroborate the results since no significant differences are observed after 9 weeks of storage. The results presented in Figure 2c show that the zeta potential of LNPs with CTAB remains between 20 and 30 mV during the 9 weeks. Likewise, LNPs functionalized with HA present zeta potential values of around 10 mV throughout the study time, showing that the functionalization remains stable. Regarding the encapsulation rate (Figure 2d), some variations in NAR@NPs_CTAB_ are observed at the end of the first week, but in the following weeks, the values tend to stabilize. Similarly, some variations are also observed in NAR@NPs_HA_ with a tendency to stabilize at the end of the 6 weeks of study. However, these variations obtained for the encapsulation rate are within the error associated with the techniques used for quantification.

**Figure 3:**
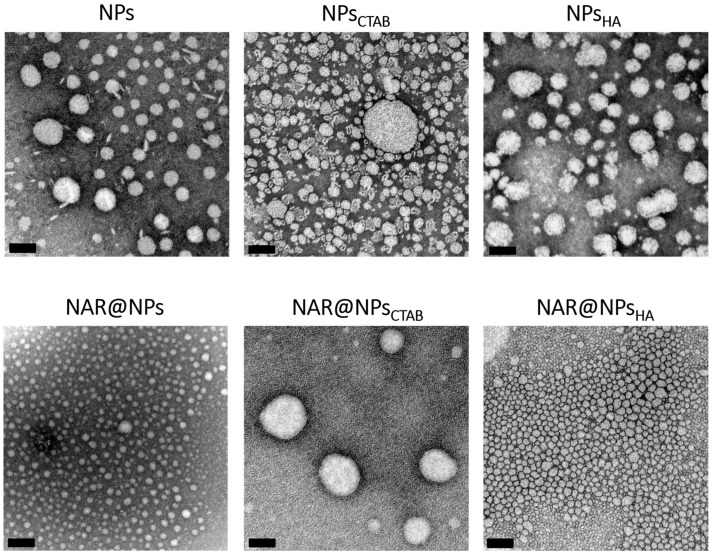
Transmission electron microscopy photographs of functionalized and non-functionalized lipid nanoparticles. Magnification 80,000×. Scale bar: 100 nm.

Nanoparticle morphology and size distribution were also evaluated using transmission electron microscopy (TEM). Figure 3 shows that all formulations present spherical morphology, and that entrapment of NAR and HA functionalization did not affect the nanoparticle’s morphology.

In this technique, the size obtained for the nanoparticles is slightly smaller than that obtained by the NTA technique, however, it is necessary to take into account that for the TEM analysis the NPs are adsorbed on the surface of the copper grid and therefore there is no influence of the layer of hydration present when we analyze the nanoparticles by the other techniques. Despite this, the similarity of the results for the PDI and the span is corroborated by the TEM analysis, where it is also verified that the formulations are uniform in terms of size. In the case of the NPs functionalized with HA, there is a slight difference in electron density around the NPs (more evident in the NPs_HA_ image), which demonstrates the success of the functionalization.

### 2.2. In Vitro Release Studies

As previously mentioned, oral administration of drugs is still the most accepted route of administration. However, the development of oral formulations still presents several challenges that are essentially related to the physicochemical properties of drugs, which include low solubility in water (0.475 mg·mL^−1^ at 25 °C [37]) and membrane permeability [38]. In addition, drugs may have low bioavailability due to barriers that are encountered in vivo, including, for example, the pH of the gastrointestinal tract and intestinal microflora [38]. Nanotechnology, associated with the development of drug delivery systems, has helped to overcome these barriers. However, when developing systems of this type, it is necessary to ensure that they will function properly in vivo. In this sense, the NAR release study was carried out in media that simulated the gastrointestinal environment (FaSSGF—pH 1.6 and FaSSIF—pH 6.5) and the physiological environment (PBS—pH 7.4). Although these are simpler solutions than human fluids, they have substances in their composition—such as lecithin, pepsin, and/or sodium taurocholate—that can modify the dissolution rate of the bioactive compounds [18,39]. Additionally, PBS (pH 7.4) was used as a way to simulate the physiological environment.

**Figure 4:**
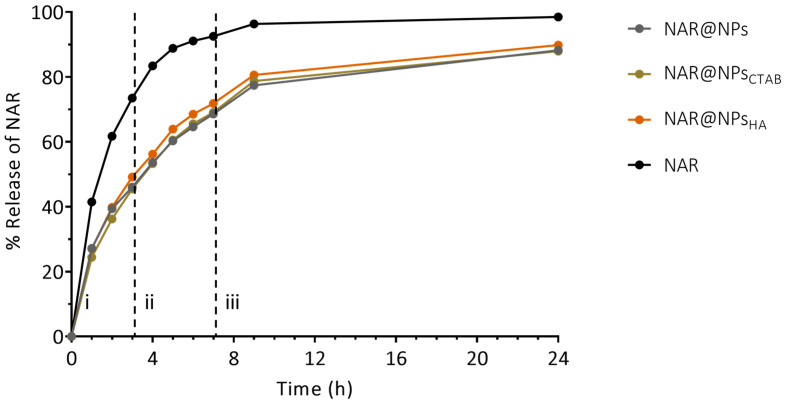
Cumulative NAR release for NAR@NPs, NAT@NPs_CTAB_, and NAR@NPs_HA_. Formulations for cumulative release profiles simulated in three conditions after oral administration. Vertical dashed lines represent mimetic medium changes: (i) gastric media, (ii) intestinal media, and (iii) physiological media. Free NAR was used as control. Values represent mean ± SD (*n* = 3).

Analyzing the results obtained (Figure 4), it is possible to observe that the amount of free NAR, in all pH values tested, is higher than the release of NAR from nanoparticles. The results show that at the end of 7 h (the end of the regime that simulates the intestinal fluid), the nanoparticles released about 70% of the NAR, while free NAR crossed the dialysis membrane more than 90%. On the other hand, at physiological pH, there is a tendency towards stabilization in the amount of NAR release. Although no clear differences are observed between the three types of nanoparticles, taken together, these results indicate that the use of LNPs for the oral administration of NAR can be a good strategy, since it will allow a greater amount of NAR to overcome the barriers of the gastrointestinal tract. This becomes advantageous since, as described above, the intestinal microflora causes the degradation of NAR [9,10,13,14,15].

The presence of functionalization with HA will be advantageous for specific targeting. It is known that HA interacts with the CD44 receptor, which is expressed in most cells [31]. It is also known that activated macrophages overexpress this receptor [40]. Therefore, the functionalization of the surface of the nanoparticles with HA will allow the targeting of inflamed cells.

The release kinetics profile of NAR was studied by applying the mathematical models above referred. Considering the values of the correlation coefficients (R^2^) obtained (Table S1) the Korsmeyer–Peppas model is the one that best describes the mechanism of NAR release at pH 1.6 (first regime) for all formulations. The Korsmeyer–Peppas equation is as follows (Equation (1)):

(1) $$\frac{M_{T}}{M_{\infty }}=kt^{n}$$

where $M_{T}/M_{\infty }$ is the fraction of NAR released at time *t*, k is the rate constant, and *n* is the release exponent. The *n*-value is used to characterize different release mechanisms [41]. The *n*-value for the release of NAR for all pH values under study is greater than 0.89, indicating that the release occurs by super case-II, here the process that controls the release mechanism is sorption, leading to the breakdown of the nanoparticle [42]. For values of pH 6.5 (second regime) the first-order model is the best fit. The first-order kinetics is indicative that drug release is dependent on the concentration [42,43].

### 2.3. Cell Viability Assays

Following ISO 10993 recommendations [44], the cytocompatibility of the developed nanoparticles was tested in the L929 fibroblast cell line (concentration range: 1.00–33.00 µg·mL^−1^). Subsequently, the THP-1 cell line was also used to assess the cytocompatibility in monocytes and macrophages (concentration range: 0.06–2.00 µg·mL^−1^).

**Figure 5:**
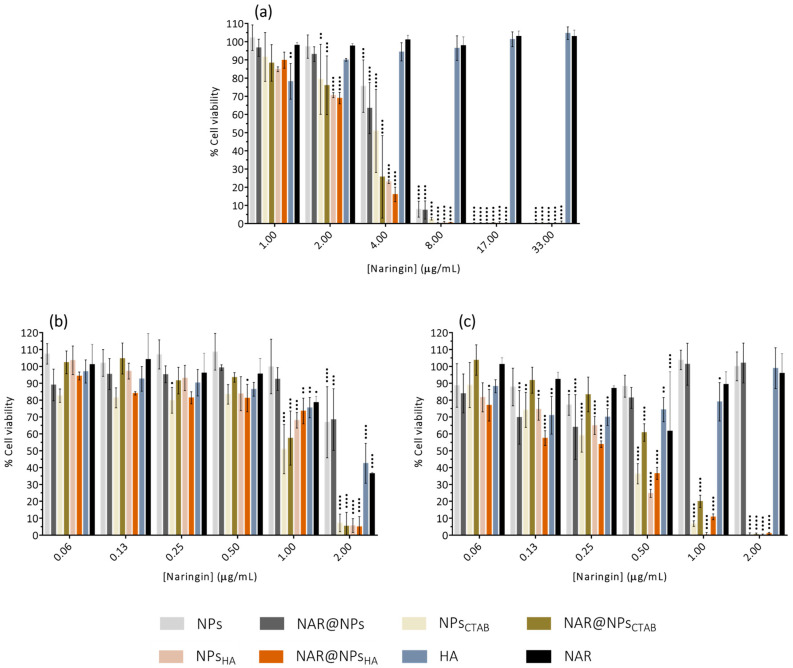
Effect of developed nanoparticles, free HA, and free NAR, at different concentrations after 24 h of incubation, on the viability of (**a**) fibroblasts (L929 cell line), (**b**) monocytes (THP-1 cell line), and (**c**) macrophages (THP-1 cell line). Data are expressed as mean ± SD (*n* = 3). Differences between groups were assessed using two-way ANOVA followed by Dunnett test. ^•^ *p* < 0.05, ^••^ *p* < 0.01, ^•••^ *p* < 0.001 and ^••••^ *p* < 0.0001 in comparison to the positive control.

Figure 5a shows that for all concentrations under study, both NAR and HA do not exert toxic effects. In the case of nanoparticles, all formulations are cytocompatible for concentrations equal to, or below 2.00 µg·mL^−1^ in NAR (corresponding to 164 µg·mL^−1^ in lipid). For all other concentrations, the viability is below 70%, which is indicative of cytotoxic effects, which are more evident for LNPs with positive zeta potential. It has already been reported that nanoparticles with a positive surface charge are more toxic than nanoparticles with a negative surface charge [45,46,47]. This difference is due to the fact that positive nanoparticles interact more efficiently with the cell membrane due to the electrostatic interactions established with the negative charges of the cell membrane (from phospholipids and proteins) [45,46,47].

Inflammation is a biological defense process of the body, where monocytes and macrophages play a key role in orchestrating an appropriate inflammatory response [48]. These cells are involved not only in the generation of inflammatory mediators [48] but also contribute to the resolution of inflammation and the restoration of homeostasis [48,49]. For this reason, dysregulation of monocytes and macrophages is often involved in the pathophysiology of chronic inflammatory and autoimmune diseases [48]. During the inflammatory process, monocytes leak from the bloodstream into the inflamed site [50,51] where they differentiate into macrophages [48,50]. Due to the importance of these cells in the inflammatory process, cell viability in the THP-1 cell line was evaluated. Based on the results obtained on the L929 cell line, the highest concentration tested on these cells was 2.00 µg·mL^−1^ in NAR.

Figure 5 shows the results obtained for cell viability in monocytes (Figure 5b) and macrophages (Figure 5c). In the case of monocytes, we verified that the concentration of 2.00 µg·mL^−1^ is the one that presents the most evident effects on toxicity, and this effect is more evident in the case of LNPs with positive zeta potential values. For lower concentrations, it appears that only NPs_CTAB_ and NPs@NAR_CTAB_ have cell viability values below 70% at a concentration of 1.00 µg·mL^−1^. In the case of the four lowest concentrations tested (0.06–0.50 µg·mL^−1^), all formulations proved to be cytocompatible. The results obtained for macrophages show that nanoparticles with positive zeta potential have very low cell viability values for concentrations 0.50–2.00 µg·mL^−1^. Concentrations below 0.50 µg·mL^−1^ are already shown to be cytocompatible. In fact, it has already been described that nanoparticles with positive zeta potential often have lower cytocompatibility [52,53], which may be due, in part, to the fact that they interact more efficiently with the negatively charged cell membrane [52]. Moghadam et al. showed that positive nanoparticles have a greater ability than negative nanoparticles to destabilize and break lipid vesicles formed by 1,2-dioleoyl-*sn*-glycero-3-phosphocholine [54].

It is also verified that the cellular models in studies present some variability between them. It is important to highlight that the cells are distinct and have different functions and locations in vivo, which certainly contributes to the observed differences.

### 2.4. Cell Toxicity Assays

It is known that cytotoxic effects are generally associated with damage to the integrity of the cell membranes [55]. The enzyme lactate dehydrogenase (LDH), present in the cytosol [56] of all cell types, [57] is rapidly released when the membrane loses integrity [56,57]. In this sense, the LDH assay was carried out to verify whether the developed LNPs cause damage to the cell membrane.

**Figure 6:**
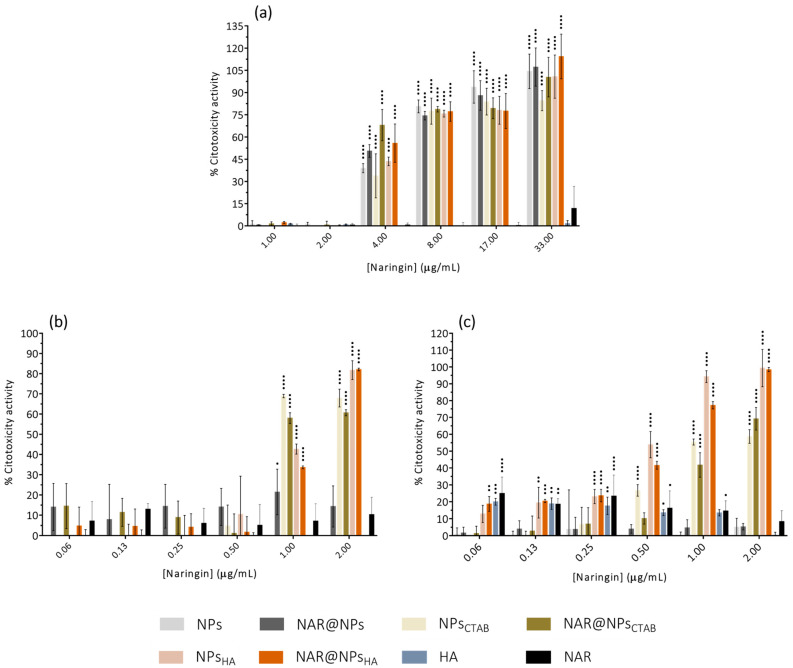
Cell cytotoxicity assessed by LDH assay for developed nanoparticles, free HA, and free NAR, at different concentrations after 24 h of incubation, on the viability of (**a**) fibroblasts (L929 cell line), (**b**) monocytes (THP-1 cell line), and (**c**) macrophages (THP-1 cell line). Data are expressed as mean ± SD (*n* = 3). Differences between groups were assessed using two-way ANOVA followed by Dunnett test. ^•^ *p* < 0.05, ^••^ *p* < 0.01, ^•••^ *p* < 0.001 and ^••••^ *p* < 0.0001 in comparison to the positive control.

The results obtained for the LDH assay with the L929 cell line (Figure 6a, concentration range: 1.00–33.00 µg·mL^−1^) show a high release of the LDH enzyme for concentrations equal to or greater than 4.00 µg·mL^−1^ in NAR for all LNPs developed. In the case of NAR and HA, there is no cytotoxic effect at any of the tested concentrations. These results are in line with the results obtained for cell viability (Figure 6a) since the highest rates of LDH enzyme release correspond to concentrations where metabolic activity was lower.

Figure 6b,c show the LDH assay results for the THP-1 cell line (concentration range: 0.06–2.00 µg·mL^−1^). Similar to what happens with the L929 cell line (Figure 6a), the highest tested concentration is the one that induces the greatest release of LDH enzyme both in monocytes (Figure 6b) and macrophages (Figure 6c). However, it turns out that this effect is more pronounced in LNPs with positive zeta potential. As previously mentioned, this type of LNP interacts more efficiently with cell membranes but also presents more toxicity [52,53]. Induction of membrane cavities may occur [54], which in turn leads to the release of the enzyme LDH.

Based on the results obtained, a concentration of 0.25 µg·mL^−1^ in NAR was used for subsequent tests. Since monocytes are differentiated into macrophages at the site of inflammation, from this stage onwards all assays were performed with cells differentiated into macrophages.

### 2.5. Hemolysis

As previously mentioned, in vivo NPs have to overcome a series of biological barriers so that they can exert the effects for which they were designed. One such barrier is the bloodstream, and in fact, blood is not only a barrier for intravenously administered nanoparticles, but it is also a gateway for nanoparticles administered by other routes to reach their target site [58]. Once in the bloodstream, NPs will interact with blood cells [59]. Therefore, when developing drug delivery systems based on NPs, it is extremely important to evaluate the interactions that may occur at the blood level as a way to reduce their toxicity and increase their effectiveness. The blood–NP interaction can be assessed for compatibility with blood cells by assessing the hemolytic potential [60].

**Figure 7:**
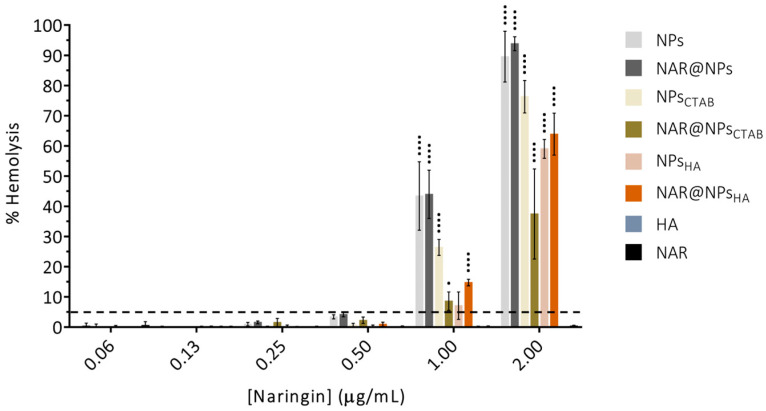
Hemolysis percentage obtained for the developed nanoparticles. Data are expressed as mean ± SD (*n* = 3). Dashed line is indicative of a hemolytic rate of 5%. Differences between groups were assessed using two-way ANOVA followed by Dunnett test. ^•^ *p* < 0.05 and ^••••^ *p* < 0.0001 in comparison to the free NAR.

Figure 7 shows the results obtained for the study of interactions between developed LNPs with red blood cells. The assay was carried out at the same concentrations used for biocompatibility studies on the THP-1 cell line (concentration range: 0.06–2.00 µg·mL^−1^), and the possible toxicity was analyzed after 1 h of contact, through the percentage of hemolysis. It was found that only concentrations of 1.00 and 2.00 µg·mL^−1^ in NAR negatively interacted with red blood cells, causing their lysis. In turn, the remaining concentrations (0.06–0.50 µg·mL^−1^) showed a percentage of hemolysis below 5%. NAR and HA did not cause hemolysis in any of the tested concentrations. According to the rate of hemolysis obtained, NPs can be classified into (i) non-hemolytic—hemolysis rate below 2%, (ii) slightly hemolytic—hemolysis rate between 2 and 5%, and (iii) hemolytic—more than 5% of hemolysis [60,61]. Nevertheless, results with hemolytic rates below 5% are considered non-toxic [62]. These results show that the developed LNPs can be a viable alternative for NAR administration. Effectively, NAR did not lead to the use of LNPs as a delivery vehicle and will allow a more controlled release and a more targeted delivery to the inflamed site, which will translate into a great advantage in clinical practice.

### 2.6. Cell Uptake and Cell Uptake Pathways Assays

**Figure 8:**
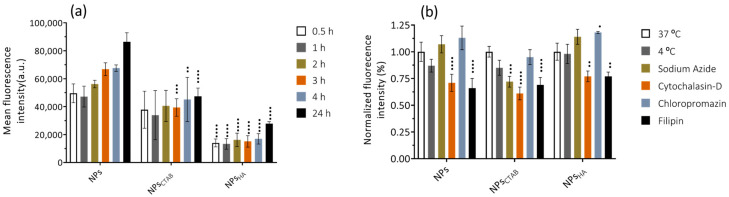
(**a**) Cellular uptake of fluorescence-marked nanoparticles in THP-1 macrophages over time (0.5, 1, 2, 3, 4, and 24 h), at a concentration of 0.25 µg·mL^−1^. Data are expressed as mean ± SD (*n* = 3). Differences between groups were assessed using two-way ANOVA followed by Tukey test. ^••^ *p* < 0.01, ^•••^ *p* < 0.001, and ^••••^ *p* < 0.0001 in comparison to the NPs. (**b**) Effect of low temperature and pathway mechanism inhibitors on the uptake of fluorescence-marked nanoparticles by THP-1 macrophages. Data are expressed as mean ± SD (*n* = 3). Differences between groups were assessed using two-way ANOVA followed by Dunnett test. ^•^ *p* < 0.05, ^••^ *p* < 0.01, ^•••^ *p* < 0.001 and ^••••^ *p* < 0.0001 in comparison to the control at 37 °C.

The results obtained for the uptake studies in macrophages (Figure 8a) show that internalization occurs after 0.5 h of contact between the cells and the LNPs (at a concentration of 0.25 µg·mL^−1^). It is described that particles with positive zeta potential are more internalized by cells than particles with negative or neutral zeta potential [63]. This happens due to the favorable interactions that occur between the positively charged particles and the negative charge of the cell membrane [46,47]. Despite this, what is verified is that the LNPs, with negative zeta potential, are internalized more quickly. When nanoparticles are exposed to biological fluids, adsorption of proteins and other biomolecules on their surface may occur, leading to the formation of the so-called protein corona that will regulate cell recognition and internalization [64,65]. In fact, the literature reports some examples where the cellular internalization of particles with a positive surface charge is lower than the internalization of negative nanoparticles. Lunov et al. report that in monocytes and macrophages (THP-1 cell line), positive nanoparticles are less efficiently internalized than negative nanoparticles. The results presented also show that the presence of serum in the medium influences the internalization, being verified as a lower internalization, compared to the test carried out in Hank’s balanced salt solution (HBSS) [66].

Between NPs_CTAB_ and NPs_HA_, it is verified that particles without functionalization are more rapidly internalized. Thinking in terms of zeta potential value, these results are in line with the literature, since NPs_CTAB_ present zeta potential values farther from neutrality. Despite this, the presence of HA on the surface of LNPs will allow targeting to activated macrophages, which overexpressed the CD44 receptor. This receptor is known to be involved in the binding, endocytosis, and metabolism of HA [67]. In addition, activation of the immune system in response to inflammation can induce HA–CD44 binding [68]. It is also reported that HA–CD44 binding during the inflammatory response is accompanied by increased expression of CD44 on the cell surface and that peak levels of HA–CD44 binding generally take between 2 to 3 days [68].

To investigate the uptake mechanism of LNPs, the assay was further performed by exposing the cells to specific inhibitors before treatment with the fluorescent marked nanoparticles. The study inhibitors were (i) chlorpromazine—clathrin-mediated endocytosis inhibitor, (ii) filipin—caveolae-mediated endocytosis, and (iii) cytochalasin-D—macropinocytosis and phagocytosis inhibitor [40,69]. To assess whether the uptake was energy dependent, the cells were also incubated at 4 °C and used sodium azide as an inhibitor [42]. The results obtained (Figure 8b) show that in the presence of cytochalasin-D and filipin, the internalization of the three types of nanoparticles is less efficient. This is indicative that the cellular internalization of LNPs is mediated by endocytosis. Filipin interacts with cholesterol present in the cell membrane and blocks caveolae-mediated endocytosis [70,71], whereas cytochalasin-D interacts with actin filaments [70,72], thus blocking all major endocytic pathways [70]. Indeed, it has been reported that positive nanoparticles are preferentially internalized by macropinocytosis [73], while negative nanoparticles are internalized by caveolae-mediated mechanisms [74]. This goes against the results obtained since, although the differences are not very evident, filipin has a greater effect on the internalization of negative nanoparticles and cytochalasin-D on positive nanoparticles.

It also verified that internalization is influenced by temperature, in the case of NPs, and by the presence of sodium azide, in the presence of NPs_CTAB_ and NPs_HA_. This corroborates the results obtained with the remaining inhibitors since endocytosis is an energy-dependent process and is therefore influenced by temperature or ATPase inhibitors, like sodium azide [75].

### 2.7. Anti-Inflammatory Activity

Inflammation is the body’s physiological response to harmful stimuli [76]. It is a coordinated cascade of events that under physiologically favorable conditions protects the body against new injuries, promoting the repair of damaged tissue [76] and restoring tissue homeostasis [48]. Despite this, when this response is not properly controlled it evolves into chronic inflammation, which plays an important role in the development, for example, of autoimmune diseases and cancer [48,77,78]. During the inflammatory response, monocytes are attracted to the inflamed site where they differentiate into macrophages, and together with resident cells, promote the resolution of inflammation [79]. In the presence of an inflammatory stimulus, macrophages secrete inflammatory mediators, such as interleukin (IL)-1β, IL-6, and tumor necrosis factor (TNF-α) [80,81], which, although they are responsible for initiating the inflammatory response, their overproductions are harmful [82].

In this sense, an assay of anti-inflammatory activity in macrophages was carried out, where lipopolysaccharide (LPS) was used as a stimulus to induce the polarization of macrophages (M0 macrophages) to an inflamed state (M1 macrophages—pro-inflammatory). Once the LNPs of greatest interest are those functionalized with HA (at a concentration of 0.25 µg·mL^−1^), and LNPs with a positive surface charge are associated with higher toxicity values, CTAB nanoparticles were not considered for the anti-inflammatory studies.

**Figure 9:**
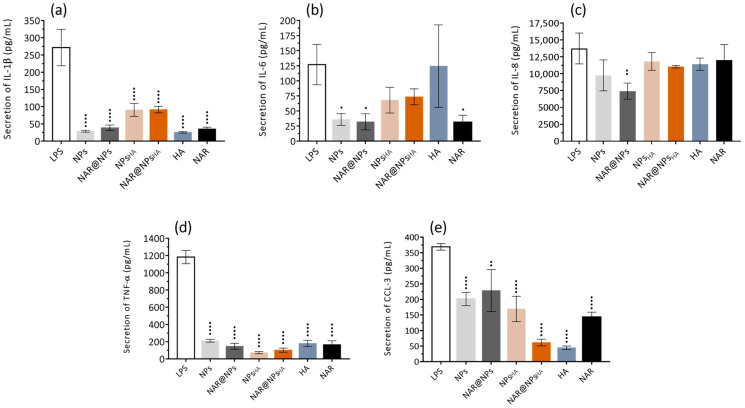
ELISA for interleukin secretion: (**a**) IL-1β, (**b**) IL-6, (**c**) IL-8, (**d**) TNF-α, and (**e**) CCL-3. Cells were pre-incubated with 0.5 µg·mL^−1^ of LNPs and controls (free NAR and free HA), for 2 h and then were stimulated to M1 macrophages with LNPs and incubated for 24 h. Data are expressed as mean ± SD (*n* = 3). Differences between groups were assessed using one-way ANOVA followed by Dunnett test. ^•^ *p* < 0.05, ^••^ *p* < 0.01 and ^••••^*p* < 0.0001 in comparison to the LPS.

The results obtained (Figure 9) show that, in general, the LNPs produced were capable of inhibiting the secretion of the inflammatory mediators under study (IL-1β, IL-6, IL-8, TNF-α, and CCL-3), however, this observation is less evident for IL-8. Analyzing the results obtained in more detail, it was observed that the expression of IL-1β, IL-6, and IL-8 is reduced, respectively, by 86, 72, and 29% (compared to LPS) after treatment with NPs. Similarly, treatment with NAR@NPs also decreases the expression of IL-1β, IL-6, and IL-8 by 86, 75, and 46%, respectively (compared to LPS). In the case of the TNF-α and CCL-3 markers, it appears that the formulations with the greatest effect are those functionalized with HA, leading to a decrease in the expression of the mediators by between 80 and 90%. It is also verified that except for inflammatory mediator CCL-3, NPs and NAR@NPs always exert a similar or more inhibitory effect than free NAR. In the case of NPs_HA_ and NAR@NPs_HA_ it is verified that their effects are less pronounced than free NAR for IL-1β and IL-6, even so, it is verified that they manage to inhibit the effects of LPS by around 66% for IL-1β and around 45% for IL-6 (compared to LPS). These results are in line with the literature that reports that NAR can act by negatively regulating the expression of IL-1β, IL-6, IL-8, and TNF-α [16,83,84]. Liu et al. show that NAR (50, 100, and 200 µM—corresponds to 29, 58, and 116 µg·mL^−1^) is able to decrease, significantly and dose-dependently, the expression of IL-8 and CCL-3 [85]. Mohanty et al. developed polymeric NPs for NAR delivery (NAR-PLGA-NPs). Studies of anti-inflammatory activity have shown that, compared to free NAR and placebo NPs, NAR-PLGA-NPs can reduce IL-6 and TNF-α, and also lead to increased production of IL-10 (anti-inflammatory cytokine) [16].

Furthermore, the results presented show that empty LNPs also have an anti-inflammatory effect. These effects may be due to the oleic acid present in the matrix of LNPs, which in itself already has an anti-inflammatory effect. Santamarina et al. report that oleic acid is capable of reducing the levels of IL-1β, IL-6, and TNF-α [86]. On the other hand, Medina-Montaro et al. show that empty lipid nanoparticles can regulate the levels of IL-1β, IL-6, and TNF-α [87]. Taken together, the results show that the use of the developed LNPs manages to regulate the release of inflammatory mediators. Although LNPs with HA do not show the most pronounced effects, the introduction of HA into the matrix does not induce a higher production of the studied mediators, so this functionalization strategy can still be advantageous to allow the targeting of activated macrophages. Several studies in the literature show the role of HA in inflammation. A detailed study is presented by Marinho et al. [31] where a survey of the role of HA as a therapeutic or supporting agent in various inflammatory pathologies is presented. Furthermore, the generally weak effect of HA or functionalized LNPs may be due to the fact that the HA–CD44 interaction reaches maximum levels only after 2 or 3 days [68], as previously mentioned, which can camouflage the results obtained for the inflammatory mediators under study.

### 2.8. Reactive Oxygen Species Assay

Reactive oxygen species (ROS) produced during the inflammatory response can induce oxidative stress [48,88]. Elevated levels of ROS can induce permanent cell damage through oxidation of DNA, RNA, proteins, and lipids [89,90], which will result in cell death or necrosis [89]. Furthermore, ROS can be triggered and lead to several inflammatory pathologies [91]. Flavonoids, as a hydrogen-donating species [92,93], can sequester ROS [92].

**Figure 10:**
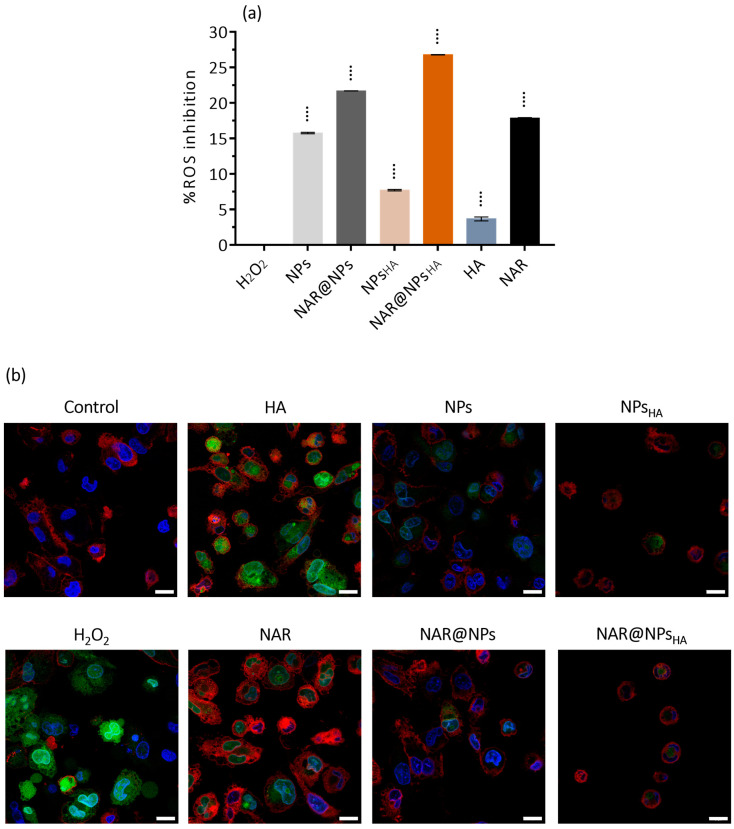
(**a**) Reactive oxygen species (ROS) scavenging activity was measured by DCFH-DA assay. Data are expressed as mean ± SD (*n* = 3). Differences between groups were assessed using one-way ANOVA followed by Dunnett test. ^••••^ *p* < 0.0001 in comparison to the H_2_O_2_. (**b**) Confocal microscopy for ROS detection. THP-1 macrophages were treated for 24 h in the absence (control) and in the presence of developed NPs, NAR, or HA. Cells were then stained for ROS (2′,7′-dichlorofluorescein diacetate—DCFH-DA, green), nucleus (Hoechst 33342, blue), and cell membrane (CellMask^TM^, red). Scale bar: 20 µm.

Through the DCFH-DA assay, we evaluated whether the developed LNPs (at a concentration of 0.25 µg·mL^−1^) provoked the production of ROS in macrophages. Figure 10a shows the inhibition of ROS production compared to the H_2_O_2_ control. What is verified is that the developed LNPs, NAR, and HA induce less ROS production than H_2_O_2_, where NAR@NPs_HA_ is more promising since it presents the highest inhibition rate. The study reported by Kim et al. shows that concentrations of 1 mM (corresponds to 580 µg·mL^−1^) of NAR can induce ROS production [94], which corroborates the fact that even in the presence of NAR our results indicate ROS production.

Figure 10b shows the results obtained for the DCFH-DA test using confocal microscopy. As evidenced by the images presented, it is verified that ROS generation occurs in the presence of LNPs, however, this production is lower than in the presence of H_2_O_2_, which is in line with the results obtained by flow cytometry. It is also verified that LNPs induce less ROS production than NAR and even HA. Therefore, the use of LNPs may be a viable alternative for the administration of NAR.

## 3. Materials and Methods

### 3.1. Materials

Gelucire^®^ 43/01 was a kind gift from Gattefossé (Gattefossé, Saint-Priest, France). Hyaluronic acid (HA, 250 kDa) was a kind gift from Bloomage Biotechnology (Bloomage Biotechnology Corporation, Jinan, China). Oleic acid and Tween^®^ 80, Triton^TM^-X-100 for molecular biology, phorbol 12-myristate 13-acetate (PMA), Dulbecco’s Phosphate Buffered Saline pH 7.4 (DPBS), sodium acetate, acetic acid, sodium chloride, resazurin sodium salt, lipopolysaccharide (LPS, from *Escherichia coli* 055:B5 strain), trypan blue solution, propidium iodide, chlorpromazine, filipin, cytochalasin-D, sodium azide, and 2′,7′-dichlorofluorescein diacetate (DCFH-DA) were obtained from Sigma-Aldrich^®^ (St. Louis, MO, USA). Naringin (≥95%) was purchased from Cayman Chemical Company (Ann Arbor, MI, USA). Absolute ethanol was acquired from Thermo Fisher Scientific (Waltham, MA, USA). Cetyltrimethylammonium bromide (CTAB) was purchased from VWR International (Leuven, Belgium). Fated state simulated gastric fluid (FaSSGF) and fasted state simulated intestinal fluid (FaSSIF) were prepared by using SIF instant power (Phares Drug Delivery AG, Muttenz, BL, Switzerland) according to the manufacturer’s instructions. L929 fibroblast cell line was obtained from European Collection of Authenticated Cell Cultures (ECACC, Salisbury, UK) (ATCC^®^ CCL-1^TM^) and THP-1 macrophage cell line (ATCC^®^ TIB-202 ^TM^) was obtained from the American Type Culture Collection (ATCC). Dulbecco’s Modified Eagle’s Medium (DMEM)+GlutaMAX^TM^, Roswell Park Memorial Institute medium (RPMI), 0.25% Trypsin–EDTA (1×), penicillin–streptomycin (Pen-Strep) and heat-inactivated fetal bovine serum (FBS) (origin: South America) were purchased from Gibco^®^ by Life Technologies^TM^ (Loughborough, Leicestershire, UK), lactate dehydrogenase (LDH) cytotoxicity detection kit was from Takara Bio Inc. (Kusatsu, Shiga, Japan). Hoechst 33342^®^ and CellMask^TM^ were purchased from Invitrogen by Thermo Fisher Scientific (Waltham, MA, USA). Ultrapure water (18.2 MΩ cm) was purified by an ultra-pure water system (Healforce, Shanghai, China).

### 3.2. Lipid Nanoparticles Synthesis

Nanostructured lipid carriers were produced by ultra-sonication method with slight modifications from the method described by Granja et al. [23]. Briefly, the lipid phase constituted by Gelucire^®^ 43/01 (200 mg) and oleic acid (70 mg) was melted at a temperature above the phase transition temperature (60 °C). The molten lipid phase was dispersed in 4.27 mL of aqueous phase (water, ethanol absolute (2.2% *v/v*), and Tween^®^80 (120 mg)) at the same temperature followed by sonication (Sonics and Material Vibra-Cell^TM^ with a CV-18 probe; Newtown CT, USA) (5 min at 80% intensity). For the synthesis of NAR-loaded LNPs (NAR@NPs), NAR (0.21% *w/v*) was added to the aqueous phase. The formulations were then stored at room temperature.

For the cellular internalization and uptake mechanism assays, fluorescent-marked non-functionalized and functionalized NPs were synthesized with 0.25% (*w/w*) of the total lipid mass of coumarin 6 added to the lipid phase.

#### HA-Functionalized LNPs

The functionalization with HA (MW: 250 kDa) was carried out by electrostatic interaction. Thus, CTAB (5 mg) was added in the lipid phase, to change the charge of the nanoparticles (NPs_CTAB_ or NAR@NPs_CTAB_). Then 1 mL of HA solution (0.02% *w/v*) was added dropwise into NPs_CTAB_ or NAR@NPs_CTAB_ under stirring (overnight) at 500 rpm at room temperature. From this point onwards, LNPs functionalized with HA are called NPs_HA_ and NAR@NPs_HA_.

### 3.3. Physicochemical Characterization

The stability of LNPs is influenced by their properties and characteristics, which may also influence their interactions with target cells and tissues [95]. Thus, the formulations developed were characterized in terms of size, polydispersity index, zeta potential, encapsulation efficiency, and morphology (through transmission electron microscopy).

#### 3.3.1. Particle Size Measurement

The produced nanoparticles were characterized by their mean hydrodynamic diameter and size distribution (polydispersity index) in a particle size analyzer (Brookhaven Instruments Corporation, Software: Particle Sizing v.5 Brookhaven Instruments; Holtsville, NY, USA), operating at a scattering angle of 90°, at 25 °C, with dust cut-off set to 30 and refractive index of the particles set to 1.33. Samples were diluted (1:100) in ultrapure water and the measurements were performed by 6 runs of 2 min each and the mean size and polydispersity index of the formulation were obtained. Nanoparticle size distribution was also determined using nanoparticle tracking analysis (NTA, NanoSight NS300, Malvern Instruments, Worcestershire, UK) equipped with a 488 nm laser. Samples were diluted at a ratio of 1:100,000 and injected using a 1 mL sterile syringe in the viewing chamber and measured under constant flow (infusion rate of 50) at 25 °C. Size measurements were obtained based on five videos of 20 s each captured with a camera level of 10 and a detection threshold of 5. For each sample, 5 size measurements were conducted. Data were analyzed using the NTA 3.4. software.

#### 3.3.2. Zeta Potential Measurement

Characterization of the zeta potential of the particles was performed in a zeta potential analyzer (ZetaPALS, Brookhaven Instruments Corporation, Software: PALS Zeta Potential Analyzer v.5, Brookhaven Instruments; Holtsville, NY, USA) operating at a scattering angle of 90°, at 25 °C. Samples were diluted (1:100) in ultrapure water and the measurement was performed by 6 runs of 10 cycles each and the zeta potential and standard deviation of the formulations were obtained.

#### 3.3.3. Encapsulation Efficiency (EE)

Formulations were diluted in ultrapure water (1:20) and centrifuged in Amicom^®^ Ultra Centrifugal Filter Devices, Ultracel^®^-50 k (50,000 NMWL) (MERK Millipore, Ltd.; Cork, Ireland). Centrifugation (Allegra^®^ X-15R Centrifuge, BECKMAN COULTER; Brea, CA, USA) was performed with spin at 2850× *g* for 20 min at 20 °C. The supernatant was discarded, and the pellet obtained was dissolved in absolute ethanol. Further, it was centrifuged at 10,000× *g* and the amount of NAR present in the supernatant was quantified by UV–Vis spectroscopy using a V-660 spectrophotometer (Jasco Corporation, Software: Spectra Manager v.2; Jasco Corporation; Easton, MD, USA) at 283 nm. A calibration curve was attained with a linearity range between 88 and 2.2 μg·mL^−1^ (R^2^ = 0.999, in ethanol).

NAR encapsulation efficiency was determined by calculating the ratio between the amount of NAR in the lipid phase and the initial amount of NAR (Equation (2): Encapsulation efficiency calculation formula):

(2) $$EE\left(\%\right)=\frac{Amount\;of\;NAR\;in\;nanoparticles}{Total\;initial\;NAR\;amount}\times 100$$

#### 3.3.4. Loading Capacity (LC)

LNPs loading capacity was calculated using the NAR@NPs encapsulation efficiency as follows (Equation (3): Loading capacity calculation formula):

(3) $$LC\left(\%\right)=\frac{EE\times Total\;initial\;NAR\;amount}{Total\;lipid\;and\;surfactant\;amount}\times 100$$

#### 3.3.5. Transmission Electron Microscopy (TEM)

Samples were prepared by placing 10 μL of the suspension (1:200 in ultrapure water) on a copper mesh grid and letting it rest for 2 min at room temperature after which the water excess was removed. For contrasting, 10 μL of 1% uranyl acetate solution was placed on the grid surface and left to rest for 30 s at room temperature. Solution excess was removed, and the samples were acquired in a JEOL 100CXII electron microscope equipped with a Gatan Orius SC200 digital camera, with an ampliation of 80,000×.

#### 3.3.6. Physical Stability Studies

LNP physical stability was assessed by monitoring for 9 weeks the particle size, PDI, zeta potential, and EE using the aforementioned methods of characterization.

### 3.4. In Vitro Release Studies under Gastrointestinal Mimetic Conditions

In vitro release studies were performed using a cellulose dialysis bag diffusion technique (Spectra/Por^®^, MWCO 6–8 kDa, Spectrum Laboratories, Rancho Dominguez, CA, USA) filled with 1 mL of the formulation.

To mimic the course of the particles in the body after oral administration, the release has been studied in media that mimic gastrointestinal conditions. For this purpose, biorelevant media were used, since the composition of such media is close to the conditions found in real organisms [96]. First, the formulations were placed in fasted-state simulated gastric fluid (FaSSGF—pH 1.6; 3 h), followed by fasted-state simulated intestinal fluid (FaSSIF—pH 6.5; 4 h), and finally, physiological pH buffer (PBS at pH 7.4; until the 24 h). All conditions were tested at 37 °C under agitation (IKA^®^-Werke RT15-P Hot Stirring Plate; Staufen, Germany), in a volume of 70 mL of buffer, and the test was performed sequentially. All conditions were tested while maintaining sink conditions.

At regular intervals, 1 mL aliquots were collected for a UV–Vis analysis and replaced by an equal amount of fresh fluid (correspondent to the one that was being used). Released NAR was analyzed by UV–Vis spectrophotometry (Jasco V-660 Spectrophotometer, Easton, MD, USA) at 283 nm.

#### Release Kinetics

The NAR@NPs release data obtained were adjusted to different mathematical models, including zero-order and first-order equations, Hixcon–Crowell, Higuchi, and Korsmeyer–Peppas models to analyze the mechanism of release of NAR@NPs and diffusion kinetics. The adjustment of each model was evaluated based on the coefficient of determination (R^2^) obtained.

### 3.5. In Vitro Celular Studies

For all in vitro cellular studies, the formulations were subjected to an additional separation step. Briefly, the formulations were diluted in ultrapure water (1:20) and centrifuged in Amicom^®^ Ultra Centrifugal Filter Devices, Ultracel^®^-50 k (50,000 NMWL) (MERK Milipore, Ltd.; Cork, Ireland). Centrifugation was performed with spin of 2850× *g* for 20 min at 20 °C. The aqueous phase was discarded, and the pellet obtained was dissolved in cell culture medium.

#### 3.5.1. Cell Culture Conditions

Fibroblasts (L929 cell line) were cultured in DMEM supplemented with 10% FBS (*v/v*) and 1% Pen-Strep (*v/v*). Cells were maintained in a 37 °C and 5% CO_2_ atmosphere (Unitherm CO_2_ Incubator 3503 Uniequip; Planegg, Germany). L929 cells were subculture at 80 to 90% confluence by chemical detachment with trypsin-EDTA and counted in a Newbauer chamber (Improved Neubauer Bright-line, Boeco; Hamburg, Germany) with 50% trypan blue solution (0.4% (*w/v*) in PBS).

Monocytes (THP-1 cell line) were cultured in RPMI-1640 supplemented with 10% FBS (*v/v*) and 1% Pen-Strep (*v/v*). Cells are maintained in a 37 °C and 5% CO_2_ atmosphere. Cells were seeded into two cell culture plates. While cells of one plate remained in suspension (monocytes), cells of another plate were differentiated (M0-macrophage) for 24 h using 20 ng·mL^−1^ of phorbol 12-myristate 13-acetate (PMA). After this incubation time, cells were treated with PMA, and the culture medium was replaced with fresh medium without PMA and incubated for 24 h in a 37 °C and 5% CO_2_ atmosphere.

#### 3.5.2. Cell Viability Assay

The effect of the NPs on cell viability was measured using the resazurin assay. L929 and THP-1 cells were seeded in a 96-well plate at a density of 5 × 10^4^ cell/well in 100 µL supplemented culture medium and incubated for 24 h at 37 °C in a 5% CO_2_ atmosphere. Different concentrations of free NAR, NAR-loaded in functionalized and non-functionalized LNPs, and correspondent amounts of placebo LNPs were added, and cells were incubated for 24 h. Positive (culture medium) and negative controls (Triton^TM^-X-100 2% (*v/v*) in DPBS) were also included. After the incubation time, plates were centrifuged at 250× *g* and room temperature (RT) for 10 min to remove cellular debris or LNPs. The supernatant was transferred to a 96-well plate and stored for the cytotoxicity assay. A 100 µL amount of resazurin solution (0.01 mg·mL^−1^ in culture medium) was then added to each cell seeded well and incubated for 2 h at 37 °C in a 5% CO_2_ atmosphere. Fluorescence was read using a microplate reader (BioTek Instrumenst Inc., Synergy HT, Software: Gen5 v1.08.4. BioTek Instruments Inc.; Winooski, VT, USA) at 560 nm excitation and 590 nm emission filter. Cell viability was determined according to the following equation (Equation (4): Cell viability calculation formula):

(4) $$Cell\;viability\left(\%\right)=\frac{Experimental\;value-Negative\;control\;value}{Positive\;control\;value-Negative\;control\;value}\times 100$$

#### 3.5.3. Cell Toxicity Assay

The cell toxicity assay analyzes the presence of lactate dehydrogenase (LDH) in the culture medium. The first steps of the LDH assay were equal to those performed on the resazurin cell viability assay until the end of the incubation period of the samples with the cells.

After supernatant centrifugation, 50 µL of the supernatant was carefully transferred to a new 96-well plate and 50 µL of the LDH Cytotoxicity Detection Kit reaction mixture was added to each well and left to incubate in the dark for 15 min at room temperature. Absorbance was then read using a microplate reader at 490 nm and 690 nm. Cytotoxicity was determined according to the following equation (Equation (5): Cell cytotoxicity calculation formula):

(5) $$Cytotoxicity\left(\%\right)=\frac{Experimental\;value-Positive\;control\;value}{Negative\;control\;value-Positive\;control\;value}\times 100$$

#### 3.5.4. Hemolysis

Human blood collected from healthy donors in ethylenediaminetetraacetic acid (EDTA)-coated tubes were kindly donated by Serviço de Hematologia from Centro Hospitalar Universitário do Porto—Hospital Santo António. Samples were centrifuged (955× *g* for 5 min, 4 °C) to separate red blood cells (RBCs). The supernatant was discarded, and the RBCs were washed three times with a sterile saline solution 0.85% (*w/v*). The RBCs obtained were diluted to 4% (*v/v*) in saline solution. Nanoformulations were diluted at the desired concentrations in saline solution. RBCs (100 µL) were then incubated with the samples (100 µL) in a 96-well plate at 37 °C for 1 h. After that, the supernatant was collected and analyzed for hemoglobin release by UV–Vis spectroscopy at 540 nm. RBCs treated with 1% Triton-X-100 were considered the positive control (100% lysis) and the RBCs treated with saline solution were considered the negative control (0% lysis). The hemolysis percentage was calculated by the following equation (Equation (6): Hemolysis calculation formula):

(6) $$Hemolysis\left(\%\right)=\frac{Experimental\;value-Negative\;control\;value}{Positive\;control\;value-Negative\;control\;value}\times 100$$

#### 3.5.5. Cell Uptake Assay

The LNP uptake into THP-1 cells (M0-macrophages) was studied by flow cytometry using the BD Accuri C6 (BD Biosciences, Erembodegem, Belgium). THP-1 cells were seeded in at the density of 1 × 10^5^ cells/well and differentiated as above mentioned.

Briefly, cells were seeded at a density of 1 × 10^5^ cells/well and differentiated as above mentioned. To study the effect of incubation time, cells were incubated with fluorescently marked non-functionalized and functionalized LNPs for 0.5, 1, 2, 3, 4, and 24 h at 37 °C with 5% CO_2_. Then, after each incubation time, cells were washed with PBS to remove any cellular debris or noninternalized LNPs. After trypsinization, cells were recovered in fresh RPMI and analyzed by flow cytometry under 488 nm excitation and 530 nm emission wavelengths. Before analysis, 1.5 µL trypan blue was added to each sample to quench the coumarin fluorescent signal coming from nanoparticles, and dead cells were excluded by staining with propidium iodide (1.5 µL). For each sample, a minimum of 10,000 events were recorded and the auto-fluorescence of cells in PBS was used as a control.

#### 3.5.6. Cell Uptake Pathway Study

Inhibition experiments to address cell uptake pathway of fluorescence-marked LNPs were performed by flow cytometry, focusing on clathrin- or caveolae-mediated endocytosis, phagocytosis, and micropinocytosis uptake mechanisms, using the method previously described by Gouveia et al. [40] and Moraes et al. [42]. Briefly, cells were seeded at a density of 1 × 10^5^ cells/well and differentiated as above mentioned. Then, cells were preincubated for 30 min at 37 °C with 5% CO_2_ with four pathways inhibitors solutions (Table S2): (i) chlorpromazine (10 µg·mL^−1^), filipin (1 µg·mL^−1^), cytochalasin-D (5 µg·mL^−1^), and sodium azide (1 µg·mL^−1^). Additionally, cells were incubated at 4 °C for 30 min. Then incubated with fluorescence-marked LNPs for 1 h at 37 °C with 5% CO_2_. Then, cells were treated for the measurement by flow cytometry as described in the cell uptake assay.

#### 3.5.7. Enzyme-Linked Immunosorbent Assay

ELISA-based methods were used to determine the concentrations of pro-inflammatory cytokines secreted into culture supernatant after lipopolysaccharide (LPS) stimulation of differentiated THP-1 cells. Firstly, THP-1 cells were seeded at a concentration of 5 × 10^4^ cells/well in a 96-well plate and differentiated as above mentioned. The following cells were pre-incubated either with free NAR, NAR-loaded in functionalized and non-functionalized LNPs, and correspondent amounts of placebo LNPs and free HA, for 2 h and then cells were stimulated to M1 macrophages with 1 µg·mL^−1^ of LPS and incubated for 24 h in a 37 °C and 5% CO_2_ atmosphere. The concentrations of the pro-inflammatory cytokines were determined in the supernatant using an ELISA kit. The analysis was completed according to the manufacturer’s protocols (R&D Systems, Minneapolis, MN, USA), for interleukin (IL)-1β (Human IL-1β DuoSet ELISA), IL-6 (Human IL-6 DuoSet ELISA), IL-8 (Human IL-8 DuoSet ELISA), CCL3 (Human CCL-3 DuoSet ELISA) and TNF-α (Human TNF-α DuoSet ELISA). Untreated cells were used as a negative control, and cells treated with LPS were used as a positive pro-inflammatory control.

#### 3.5.8. Reactive Oxygen Species Assay

Intracellular reactive oxygen species (ROS) induction due to the contact with the developed LNPs was measured by 2′,7′-dichlorofluorescein diacetate (DCFH-DA) assay. THP-1 cells were seeded at a concentration of 1 × 10^5^ cells/well and differentiated as above mentioned. Cells were incubated with LNPs for 24 h in a 37 °C and 5% CO_2_ atmosphere. After this, the LNPs were removed, the cells washed with DPBS, incubated with 10 µM of DCFH-DA for 30 min, and prepared for analysis by flow cytometry according to the method described above. Hydrogen peroxide (H_2_O_2_) was used as an ROS-positive control, incubated for 1 h before the addition of DCFH-DA.

Additionally, cellular imaging was performed using confocal laser scanning microscopy (CLSM). For this, the cells were prepared and treated as described for the assay by flow cytometry. After incubation with DCFH-DA, cells were incubated with Cell Mask^TM^ (1:1000 *v/v*), for the membrane staining. After 10 min they were washed again with PBS and fixed using Formalin solution. The nucleus was stained using Hoechst 33342^®^, (8 µM in DPBS, 10 min). Images of cancer cell internalization were acquired on a Leica Stellaris 8 confocal microscope (LeicaMicrosystems, Wetzlar, Germany) equipped with the Leica Application Suite X package (LAS X) using a λ_ex_/λ_em_ of 350⁄461 nm (Hoechst 33342^®^), λ_ex/_λ_em_ of 649/666 nm (CellMask^TM^) and λ_ex_/λ_em_ of  485/530 nm (DCFH-DA) with a resolution of 1024 × 1024 using a 63×/1.4 oil immersion objective. Images were analyzed using Fiji ImageJ software (version 2.0, National Institutes of Health, Bethesda, MD, USA). CLSM studies were performed at the Imaging by confocal and fluorescence lifetime laboratory, CEMUP, Portugal.

### 3.6. Statistical Analysis

GraphPad Prism 8 Software (San Diego, CA, USA) was used to perform the statistical analysis. Statistical comparisons of the mean between groups were assessed by one-way or two-way ANOVA. Differences were considered significant with a *p*-value under 0.05. Data are represented as mean ± SD.

## 4. Conclusions

In the present work, LNPs were developed for the targeted delivery of NAR to activated macrophages, for which the functionalization of NPs with HA was considered because activated macrophages highly present in the inflamed site overexpress the CD44 receptor. The results obtained show that although the developed NPs present an encapsulation rate of around 30–40%, this is sufficient to achieve a therapeutic effect. We also show that it is possible to achieve the sustained release of NAR over 24 h (under gastrointestinal conditions), which inevitably, in vivo, will lead to more potent biological effects than the administration of free NAR. It is also verified that the developed LNPs do not present toxicity for macrophages at concentrations of 0.25 µg·mL^−1^ in NAR and that this concentration is capable of inhibiting the release of inflammatory mediators relevant to chronic inflammatory conditions, including IL-1β, IL-6, and TNF-α. Although there are no very significant differences between empty LNPs and those loaded with NAR for the levels of the inflammatory mediators under study, it is necessary to take into account that, despite presenting therapeutic effects, NAR is present in a reduced concentration (0.25 µg·mL^−1^), which could camouflage the potential of LNPs with vehicles.

Taken together, the results show that the use of LNPs for NAR administration will be an asset. Since in the inflammatory process, surrounding monocytes are recruited and differentiated into macrophages, nanoparticles functionalized with HA are the most advantageous. As previously mentioned, these cells expressed the CD44 receptor for which HA has affinity, so its presence will be essential for active targeting. Despite this, it should be noted that the expression of this receptor accompanies the progression of the inflammatory process and that the maximum peak of HA–CD44 interaction only occurs at the end of 2–3 days. Furthermore, HA is a polymer naturally produced by the body, which will mean that LNPs are not recognized as a foreign material by the body, thus achieving a longer circulation time free from opsonization until they reach the site of action.

## Figures and Tables

**Table 1:** Physicochemical properties of the developed LNPs by DLS. Values as expressed as mean ± deviation (*n* = 3).

	Size (nm)	PDI	Zeta Potential (mV)	EE%	LC%
NPs	406.3 ± 11.9	0.060 ± 0.030	−25.82 ± 1.52	-	-
NAR@NPs	421.2 ± 11.7	0.147 ± 0.038	−25.31 ± 1.45	30.1 ± 2.9	0.7 ± 0.1
NPs_CTAB_	151.7 ± 3.5	0.156 ± 0.013	24.18 ±1.12	-	-
NAR@NPs_CTAB_	121.4 ± 2.1	0.208 ± 0.070	28.66 ± 1.70	42.6 ± 5.5	0.9 ± 0.1
NPs_HA_	163.9 ± 4.8	0.197 ± 0.020	9.06 ± 0.55	-	-
NAR@NPs_HA_	123.4 ± 3.4	0.245 ± 0.040	9.94 ± 1.72	34.2 ± 3.3	0.8 ± 0.1

PDI: polydispersity index; EE: encapsulation efficiency; LC: loading capacity.

## Data Availability

Data are contained within the article and Supplementary Materials.

## Supplementary Materials

The following supporting information can be downloaded at: https://www.mdpi.com/article/10.3390/ijms25084152/s1.

## References

[1] Bacanlı M., Başaran A.A., Başaran N., Watson R.R., Preedy V.R., Zibadi S. (2018). Chapter 4—The Major Flavonoid of Grapefruit: Naringin. Polyphenols: Prevention and Treatment of Human Disease.

[2] Ribeiro I.A., Rocha J., Sepodes B., Mota-Filipe H., Ribeiro M.H. (2008). Effect of naringin enzymatic hydrolysis towards naringenin on the anti-inflammatory activity of both compounds. J. Mol. Catal. B Enzym..

[3] Yu K.E., Alder K.D., Morris M.T., Munger A.M., Lee I., Cahill S.V., Kwon H.-K., Back J., Lee F.Y. (2020). Re-appraising the potential of naringin for natural, novel orthopedic biotherapies. Ther. Adv. Musculoskelet. Dis..

[4] Zhang L., Song L., Zhang P., Liu T., Zhou L., Yang G., Lin R., Zhang J. (2015). Solubilities of Naringin and Naringenin in Different Solvents and Dissociation Constants of Naringenin. J. Chem. Eng. Data.

[5] Zhao Y., Li Z., Wang W., Zhang H., Chen J., Su P., Liu L., Li W. (2016). Naringin Protects Against Cartilage Destruction in Osteoarthritis Through Repression of NF-κB Signaling Pathway. Inflammation.

[6] Chen R., Qi Q.-L., Wang M.-T., Li Q.-Y. (2016). Therapeutic potential of naringin: An overview. Pharm. Biol..

[7] Zhao Y., Liu S. (2021). Bioactivity of naringin and related mechanisms. Pharmazie.

[8] Akanksha S., Divya J., Vikram S., Geeta R. (2014). Naringin a Potent Antioxidant Used as Bioavailibility Enhancer for Terbinafine Hydrochloride. J. Appl. Pharm. Res..

[9] Zeng X., Su W., Zheng Y., He Y., He Y., Rao H., Peng W., Yao H. (2019). Pharmacokinetics, Tissue Distribution, Metabolism, and Excretion of Naringin in Aged Rats. Front. Pharmacol..

[10] Bharti S., Rani N., Krishnamurthy B., Arya D.S. (2014). Preclinical evidence for the pharmacological actions of naringin: A review. Planta Medica.

[11] Viswanatha G.L., Shylaja H., Moolemath Y. (2017). The beneficial role of Naringin- a citrus bioflavonoid, against oxidative stress-induced neurobehavioral disorders and cognitive dysfunction in rodents: A systematic review and meta-analysis. Biomed. Pharmacother..

[12] Lin C.-H., Chen C.-H., Lin Z.-C., Fang J.-Y. (2017). Recent advances in oral delivery of drugs and bioactive natural products using solid lipid nanoparticles as the carriers. J. Food Drug Anal..

[13] Bilia A., Isacchi B., Righeschi C., Guccione C., Maria C., Bergonzi M. (2014). Flavonoids Loaded in Nanocarriers: An Opportunity to Increase Oral Bioavailability and Bioefficacy. Food Nutr. Sci..

[14] Alam M.A., Subhan N., Rahman M.M., Uddin S.J., Reza H.M., Sarker S.D. (2014). Effect of Citrus Flavonoids, Naringin and Naringenin, on Metabolic Syndrome and Their Mechanisms of Action. Adv. Nutr..

[15] Suseem S., Joseph D. (2019). The Myth and the fact on Naringin-A Review. Res. J. Pharm. Technol..

[16] Mohanty S., Konkimalla V.B., Pal A., Sharma T., Si S.C. (2021). Naringin as Sustained Delivery Nanoparticles Ameliorates the Anti-inflammatory Activity in a Freund’s Complete Adjuvant-Induced Arthritis Model. ACS Omega.

[17] Yen F.-L., Wu T.-H., Lin L.-T., Cham T.-M., Lin C.-C. (2009). Naringenin-Loaded Nanoparticles Improve the Physicochemical Properties and the Hepatoprotective Effects of Naringenin in Orally-Administered Rats with CCl_4_-Induced Acute Liver Failure. Pharm. Res..

[18] Mitchell M.J., Billingsley M.M., Haley R.M., Wechsler M.E., Peppas N.A., Langer R. (2021). Engineering precision nanoparticles for drug delivery. Nat. Rev. Drug Discov..

[19] Patra J.K., Das G., Fraceto L.F., Campos E.V.R., Rodriguez-Torres M.d.P., Acosta-Torres L.S., Diaz-Torres L.A., Grillo R., Swamy M.K., Sharma S. (2018). Nano based drug delivery systems: Recent developments and future prospects. J. Nanobiotechnology.

[20] Wang W., Liu Q., Liang X., Kang Q., Wang Z. (2022). Protective role of naringin loaded solid nanoparticles against aflatoxin B1 induced hepatocellular carcinoma. Chem.-Biol. Interact..

[21] Imam S.S., Gilani S.J., Zafar A., Jumah M.N.b., Ali R., Ahmed M.M., Alshehri S. (2022). Preparation and Optimization of Naringin Oral Nanocarrier: In Vitro Characterization and Antibacterial Activity. Coatings.

[22] Pleguezuelos-Villa M., Mir-Palomo S., Díez-Sales O., Buso M.A.O.V., Sauri A.R., Nácher A. (2018). A novel ultradeformable liposomes of Naringin for anti-inflammatory therapy. Colloids Surf. B Biointerfaces.

[23] Granja A., Lima-Sousa R., Alves C.G., de Melo-Diogo D., Pinheiro M., Sousa C.T., Correia I.J., Reis S. (2021). Mitoxantrone-loaded lipid nanoparticles for breast cancer therapy—Quality-by-design approach and efficacy assessment in 2D and 3D in vitro cancer models. Int. J. Pharm..

[24] Lopes-de-Araújo J., Neves A.R., Gouveia V.M., Moura C.C., Nunes C., Reis S. (2016). Oxaprozin-Loaded Lipid Nanoparticles towards Overcoming NSAIDs Side-Effects. Pharm. Res..

[25] Neves A.R., Lúcio M., Martins S., Lima J.L., Reis S. (2013). Novel resveratrol nanodelivery systems based on lipid nanoparticles to enhance its oral bioavailability. Int. J. Nanomed..

[26] Kestens V., Bozatzidis V., De Temmerman P.J., Ramaye Y., Roebben G. (2017). Validation of a particle tracking analysis method for the size determination of nano- and microparticles. J. Nanopart. Res.

[27] Rasmussen M.K., Pedersen J.N., Marie R. (2020). Size and surface charge characterization of nanoparticles with a salt gradient. Nat. Commun..

[28] Smith M.C., Crist R.M., Clogston J.D., McNeil S.E. (2017). Zeta potential: A case study of cationic, anionic, and neutral liposomes. Anal. Bioanal. Chem..

[29] Németh Z., Csóka I., Semnani Jazani R., Sipos B., Haspel H., Kozma G., Kónya Z., Dobó D.G. (2022). Quality by Design-Driven Zeta Potential Optimisation Study of Liposomes with Charge Imparting Membrane Additives. Pharmaceutics.

[30] Dhiman N., Awasthi R., Sharma B., Kharkwal H., Kulkarni G.T. (2021). Lipid Nanoparticles as Carriers for Bioactive Delivery. Front. Chem..

[31] Marinho A., Nunes C., Reis S. (2021). Hyaluronic Acid: A Key Ingredient in the Therapy of Inflammation. Biomolecules.

[32] Bayer I.S. (2020). Hyaluronic Acid and Controlled Release: A Review. Molecules.

[33] Shen H., Shi S., Zhang Z., Gong T., Sun X. (2015). Coating Solid Lipid Nanoparticles with Hyaluronic Acid Enhances Antitumor Activity against Melanoma Stem-like Cells. Theranostics.

[34] Zhang X., Wan L., Li L., Xu Z., Su J., Li B., Huang J. (2018). Effects of magnetic fields on the enzymatic synthesis of naringin palmitate. RSC Adv..

[35] Zhang X., Li L., Xu Z., Liang Z., Su J., Huang J., Li B. (2013). Investigation of the Interaction of Naringin Palmitate with Bovine Serum Albumin: Spectroscopic Analysis and Molecular Docking. PLoS ONE.

[36] Almeida V.M., Branco C.R.C., Assis S.A., Vieira I.J.C., Braz-Filho R., Branco A. (2012). Synthesis of naringin 6”-ricinoleate using immobilized lipase. Chem. Cent. J..

[37] Alhalmi A., Amin S., Beg S., Al-Salahi R., Mir S.R., Kohli K. (2022). Formulation and optimization of naringin loaded nanostructured lipid carriers using Box-Behnken based design: In vitro and ex vivo evaluation. J. Drug Deliv. Sci. Technol..

[38] Alqahtani M.S., Kazi M., Alsenaidy M.A., Ahmad M.Z. (2021). Advances in Oral Drug Delivery. Front. Pharmacol..

[39] Simancas-Herbada R., Fernández-Carballido A., Aparicio Blanco J., Slowing K., Rubio Retama J., López-Cabarcos E., Torres-Suarez A. (2020). Controlled Release of Highly Hydrophilic Drugs from Novel Poly(Magnesium Acrylate) Matrix Tablets. Pharmaceutics.

[40] Gouveia V., Lopes-de-Araújo J., Costa Lima S., Nunes C., Reis S. (2018). Hyaluronic acid-conjugated pH-sensitive liposomes for targeted delivery of prednisolone on rheumatoid arthritis therapy. Nanomedicine.

[41] Singhvi G., Singh M. (2011). Review: In vitro Drug Release Characterization Models. Int. J. Pharm. Stud. Res..

[42] Moraes S., Marinho A., Lima S., Granja A., Araújo J.P., Reis S., Sousa C.T., Nunes C. (2021). Targeted nanostructured lipid carriers for doxorubicin oral delivery. Int. J. Pharm..

[43] Talevi A., Ruiz M.E. (2021). Drug Release. The ADME Encyclopedia: A Comprehensive Guide on Biopharmacy and Pharmacokinetics.

[44] (2009). Biological Evaluation of Medical Devices. Part 5: Tests for In Vitro Cytotoxicity.

[45] Weiss M., Fan J., Claudel M., Sonntag T., Didier P., Ronzani C., Lebeau L., Pons F. (2021). Density of surface charge is a more predictive factor of the toxicity of cationic carbon nanoparticles than zeta potential. J. Nanobiotechnol..

[46] Adabi M., Naghibzadeh M., Adabi M., Zarrinfard M.A., Esnaashari S.S., Seifalian A.M., Faridi-Majidi R., Tanimowo Aiyelabegan H., Ghanbari H. (2017). Biocompatibility and nanostructured materials: Applications in nanomedicine. Artif. Cells Nanomed. Biotechnol..

[47] Forest V., Pourchez J. (2017). Preferential binding of positive nanoparticles on cell membranes is due to electrostatic interactions: A too simplistic explanation that does not take into account the nanoparticle protein corona. Mater. Sci. Eng. C.

[48] Austermann J., Roth J., Barczyk-Kahlert K. (2022). The Good and the Bad: Monocytes’; and Macrophages’; Diverse Functions in Inflammation. Cells.

[49] Chang-Hoon L., Eun Young C. (2018). Macrophages and Inflammation. J. Rheum. Dis..

[50] Kratofil R.M., Kubes P., Deniset J.F. (2017). Monocyte Conversion During Inflammation and Injury. Arterioscler. Thromb. Vasc. Biol..

[51] Yang J., Zhang L., Yu C., Yang X.-F., Wang H. (2014). Monocyte and macrophage differentiation: Circulation inflammatory monocyte as biomarker for inflammatory diseases. Biomark. Res..

[52] Contini C., Schneemilch M., Gaisford S., Quirke N. (2018). Nanoparticle–membrane interactions. J. Exp. Nanosci..

[53] Zhang Y., Dahal U., Feng Z.V., Rosenzweig Z., Cui Q., Hamers R.J. (2021). Influence of Surface Ligand Molecular Structure on Phospholipid Membrane Disruption by Cationic Nanoparticles. Langmuir.

[54] Moghadam B.Y., Hou W.-C., Corredor C., Westerhoff P., Posner J.D. (2012). Role of Nanoparticle Surface Functionality in the Disruption of Model Cell Membranes. Langmuir.

[55] Zou Y., Kim D., Yagi M., Yamasaki Y., Kurita J., Iida T., Matsuyama Y., Yamaguchi K., Oda T. (2013). Application of LDH-Release Assay to Cellular-Level Evaluation of the Toxic Potential of Harmful Algal Species. Biosci. Biotechnol. Biochem..

[56] Özlem Sultan A., Marcelo L.L., Sonia S. (2017). In Vitro Cytotoxicity and Cell Viability Assays: Principles, Advantages, and Disadvantages. Genotoxicity.

[57] Karlsson H.L., Cronholm P., Hedberg Y., Tornberg M., De Battice L., Svedhem S., Wallinder I.O. (2013). Cell membrane damage and protein interaction induced by copper containing nanoparticles—Importance of the metal release process. Toxicology.

[58] de la Harpe K.M., Kondiah P.P.D., Choonara Y.E., Marimuthu T., du Toit L.C., Pillay V. (2019). The Hemocompatibility of Nanoparticles: A Review of Cell-Nanoparticle Interactions and Hemostasis. Cells.

[59] Sobot D., Mura S., Couvreur P., Kobayashi S., Müllen K. (2015). Nanoparticles: Blood Components Interactions. Encyclopedia of Polymeric Nanomaterials.

[60] Aula S., Lakkireddy S., Jamil K., Kapley A., Swamy A.V.N., Lakkireddy H.R. (2015). Biophysical, biopharmaceutical and toxicological significance of biomedical nanoparticles. RSC Adv..

[61] Weber M., Steinle H., Golombek S., Hann L., Schlensak C., Wendel H.P., Avci-Adali M. (2018). Blood-Contacting Biomaterials: In Vitro Evaluation of the Hemocompatibility. Front. Bioeng. Biotechnol..

[62] Golla K., Cherukuvada B., Ahmed F., Kondapi A.K. (2012). Efficacy, Safety and Anticancer Activity of Protein Nanoparticle-Based Delivery of Doxorubicin through Intravenous Administration in Rats. PLoS ONE.

[63] Forest V., Cottier M., Pourchez J. (2015). Electrostatic interactions favor the binding of positive nanoparticles on cells: A reductive theory. Nano Today.

[64] Yan Y., Gause K.T., Kamphuis M.M.J., Ang C.-S., O’Brien-Simpson N.M., Lenzo J.C., Reynolds E.C., Nice E.C., Caruso F. (2013). Differential Roles of the Protein Corona in the Cellular Uptake of Nanoporous Polymer Particles by Monocyte and Macrophage Cell Lines. ACS Nano.

[65] Francia V., Yang K., Deville S., Reker-Smit C., Nelissen I., Salvati A. (2019). Corona Composition Can Affect the Mechanisms Cells Use to Internalize Nanoparticles. ACS Nano.

[66] Lunov O., Syrovets T., Loos C., Beil J., Delacher M., Tron K., Nienhaus G.U., Musyanovych A., Mailänder V., Landfester K. (2011). Differential Uptake of Functionalized Polystyrene Nanoparticles by Human Macrophages and a Monocytic Cell Line. ACS Nano.

[67] Vachon E., Martin R., Plumb J., Kwok V., Vandivier R.W., Glogauer M., Kapus A., Wang X., Chow C.-W., Grinstein S. (2006). CD44 is a phagocytic receptor. Blood.

[68] Lee-Sayer S.S.M., Dong Y., Arif A.A., Olsson M., Brown K.L., Johnson P. (2015). The Where, When, How, and Why of Hyaluronan Binding by Immune Cells. Front. Immunol..

[69] Jain A.K., Thareja S. (2019). In vitro and in vivo characterization of pharmaceutical nanocarriers used for drug delivery. Artif. Cells Nanomed. Biotechnol..

[70] Porkoláb G., Mészáros M., Tóth A., Szecskó A., Harazin A., Szegletes Z., Ferenc G., Blastyák A., Mátés L., Rákhely G. (2020). Combination of Alanine and Glutathione as Targeting Ligands of Nanoparticles Enhances Cargo Delivery into the Cells of the Neurovascular Unit. Pharmaceutics.

[71] Zaki N.M., Nasti A., Tirelli N. (2011). Nanocarriers for Cytoplasmic Delivery: Cellular Uptake and Intracellular Fate of Chitosan and Hyaluronic Acid-Coated Chitosan Nanoparticles in a Phagocytic Cell Model. Macromol. Biosci..

[72] Kuhn D.A., Vanhecke D., Michen B., Blank F., Gehr P., Petri-Fink A., Rothen-Rutishauser B. (2014). Different endocytotic uptake mechanisms for nanoparticles in epithelial cells and macrophages. Beilstein. J. Nanotechnol..

[73] Foroozandeh P., Aziz A.A. (2018). Insight into Cellular Uptake and Intracellular Trafficking of Nanoparticles. Nanoscale Res. Lett..

[74] Mazumdar S., Chitkara D., Mittal A. (2021). Exploration and insights into the cellular internalization and intracellular fate of amphiphilic polymeric nanocarriers. Acta Pharm. Sin. B.

[75] Shi J.-M., Zhu L., Lan X., Zhao D.-W., He Y.-J., Sun Z.-Q., Wu D., Li H.-Y. (2020). Endocytosis Is a Key Mode of Interaction between Extracellular *β*-Amyloid and the Cell Membrane. Biophys. J..

[76] Fujiwara N., Kobayashi K. (2005). Macrophages in inflammation. Curr. Drug Targets Inflamm. Allergy.

[77] Deng Z., Liu S. (2021). Inflammation-responsive delivery systems for the treatment of chronic inflammatory diseases. Drug Deliv. Transl. Res..

[78] Ronchetti S., Migliorati G., Bruscoli S., Riccardi C. (2018). Defining the role of glucocorticoids in inflammation. Clin. Sci..

[79] Viola A., Munari F., Sánchez-Rodríguez R., Scolaro T., Castegna A. (2019). The Metabolic Signature of Macrophage Responses. Front. Immunol..

[80] Arango Duque G., Descoteaux A. (2014). Macrophage Cytokines: Involvement in Immunity and Infectious Diseases. Front. Immunol..

[81] Gabay C. (2006). Interleukin-6 and chronic inflammation. Arthritis Res. Ther..

[82] Ng P.C., Li K., Wong R.P.O., Chui K., Wong E., Li G., Fok T.F. (2003). Proinflammatory and anti-inflammatory cytokine responses in preterm infants with systemic infections. Arch. Dis. Child.-Fetal Neonatal Ed..

[83] Deenonpoe R., Prayong P., Thippamom N., Meephansan J., Na-Bangchang K. (2019). Anti-inflammatory effect of naringin and sericin combination on human peripheral blood mononuclear cells (hPBMCs) from patient with psoriasis. BMC Complement. Altern. Med..

[84] Ghanbari-Movahed M., Jackson G., Farzaei M.H., Bishayee A. (2021). A Systematic Review of the Preventive and Therapeutic Effects of Naringin Against Human Malignancies. Front. Pharmacol..

[85] Liu Y., Su W.-W., Wang S., Li P.-B. (2012). Naringin inhibits chemokine production in an LPS-induced RAW 264.7 macrophage cell line. Mol. Med. Rep..

[86] Santamarina A.B., Pisani L.P., Baker E.J., Marat A.D., Valenzuela C.A., Miles E.A., Calder P.C. (2021). Anti-inflammatory effects of oleic acid and the anthocyanin keracyanin alone and in combination: Effects on monocyte and macrophage responses and the NF-κB pathway. Food Funct..

[87] Medina-Montano C., Rivero Berti I., Gambaro R.C., Limeres M.J., Svensson M., Padula G., Chain C.Y., Cisneros J.S., Castro G.R., Grabbe S. (2022). Nanostructured Lipid Carriers Loaded with Dexamethasone Prevent Inflammatory Responses in Primary Non-Parenchymal Liver Cells. Pharmaceutics.

[88] Zuo L., Prather E.R., Stetskiv M., Garrison D.E., Meade J.R., Peace T.I., Zhou T. (2019). Inflammaging and Oxidative Stress in Human Diseases: From Molecular Mechanisms to Novel Treatments. Int. J. Mol. Sci..

[89] Checa J., Aran J.M. (2020). Reactive Oxygen Species: Drivers of Physiological and Pathological Processes. J. Inflamm. Res..

[90] Herb M., Schramm M. (2021). Functions of ROS in Macrophages and Antimicrobial Immunity. Antioxidants.

[91] El-desoky A.H., Abdel-Rahman R.F., Ahmed O.K., El-Beltagi H.S., Hattori M. (2018). Anti-inflammatory and antioxidant activities of naringin isolated from Carissa carandas L.: In vitro and in vivo evidence. Phytomedicine.

[92] Inês Amaro M., Rocha J., Vila-Real H., Eduardo-Figueira M., Mota-Filipe H., Sepodes B., Ribeiro M.H. (2009). Anti-inflammatory activity of naringin and the biosynthesised naringenin by naringinase immobilized in microstructured materials in a model of DSS-induced colitis in mice. Food Res. Int..

[93] Drummond N.J., Davies N.O., Lovett J.E., Miller M.R., Cook G., Becker T., Becker C.G., McPhail D.B., Kunath T. (2017). A synthetic cell permeable antioxidant protects neurons against acute oxidative stress. Sci. Rep..

[94] Kim H., Lee D.G. (2021). Naringin-generated ROS promotes mitochondria-mediated apoptosis in Candida albicans. IUBMB Life.

[95] Albuquerque J., Moura C.C., Sarmento B., Reis S. (2015). Solid Lipid Nanoparticles: A Potential Multifunctional Approach towards Rheumatoid Arthritis Theranostics. Molecules.

[96] Upadhyaya S.K. (2009). New And Emerging Therapies For Rheumatoid Arthritis. Apollo Med..

