# Supplementary material for: Empowering Naringin’s Anti-Inflammatory Effects through Nanoencapsulation
Source: Int J Mol Sci. 2024 Apr 9;25(8):4152. doi: 10.3390/ijms25084152 (PMC11050564; doi:10.3390/ijms25084152)
Supplement: Supplementary file 1 [file ijms-25-04152-s001.zip › ijms-2905200-supplementary.pdf]

Supplementary Material

Empowering Naringin´s Anti-Inflammatory Effects through Nanoencapsulation

Andreia Marinho [1,2], Catarina Leal Seabra [1], Sofia A. C. Lima [3], Alexandre Lobo-da-Cunha [4], Salette Reis [1], Cláudia Nunes [1,3,*]

[1]LAQV, REQUIMTE, Faculdade de Farmácia, Universidade do Porto, R. Jorge de Viterbo Ferreira 228, 4050-313 Porto, Portugal

[2]LAQV, REQUIMTE, Faculdade de Ciências, Universidade do Porto, R. do Campo Alegre s/n, 4169-007 Porto, Portugal

[3]LAQV, REQUIMTE, Instituto de Ciências Biomédicas Abel Salazar, Universidade do Porto, R. Jorge de Viterbo Ferreira 228, 4050-313 Porto, Portugal

[4]Departamento de Microscopia, Instituto de Ciências Biomédicas Abel Salazar, Universidade do Porto, R. Jorge de Viterbo Ferreira 228, 4050-313 Porto, Portugal

*Correspondence: cdnunes@ff.up.pt

1. In vitro release studies

Table S1 lists the results obtained for the release kinetics studies with an indication of the correlation coefficient values and the constants obtained for each model under study.

Table S1: Correlation coefficient (R^2) for the release of NAR at different pH values, according to the models studied.

	Regime	Zero-order		First-order		Hixson-Crowell		Higuchi		Korsmeyer-Peppas	
		R^2	k_0	R^2	k_1	R^2	k_{HC}	R^2	k_H	R^2	k_{KP}
**NAR@NPs**	**i**	0.997	0.011	0.978	0.305	0.973	-9.449	0.9852	0.053	**†1.000**	1.279
	**ii**	0.991	0.029	**†0.998**	0.054	0.983	-4.914	0.9801	0.015	0.944	0.261
	**iii**	1.000	0.000	1.000	0.002	1.000	-0.492	1.000	0.002	1.000	0.041
**NAR@NPs$_{CTAB}$**	**i**	0.996	0.012	0.981	0.316	0.995	-10.555	0.981	0.032	**†1.000**	1.320
	**ii**	0.992	0.006	**†0.999**	0.060	0.975	-5.246	0.964	0.017	0.947	0.287
	**iii**	1.000	0.000	1.000	0.002	1.000	-0.419	1.000	0.002	1.000	0.040
**NAR@NPs$_{HA}$**	**i**	0.997	0.011	0.980	0.313	0.992	-11.064	0.983	0.028	**†1.000**	1.309
	**ii**	0.992	0.004	**†0.999**	0.055	0.963	-5.148	0.966	0.013	0.947	0.263
	**iii**	1.000	0.000	1.000	0.002	1.000	-0.421	1.000	0.001	1.000	0.037

† Represents models that best fit each release profile.

k_0: zero-order constant; k_1: first-order constant; k_{HC}: release constant of Hixcon-Crowell; k_H: release constant of Higuchi; k_{KP}: release constant of Korsmeyer-Peppas.

2. Cell uptake pathway inhibitors solutions

Table S2 lists the receptors used to study the internalization pathways of the development nanoparticles, as well as their respective functions.

Table S2: Selected inhibitors and respective functions applied to mechanistic studies of nanoparticles transport by THP-1 macrophages.

	**Inhibitor**	**Concentration**	**Function**
**Endocytosis inhibitors**	Chlorpromazine	10 $\mu g \cdot mL^{-1}$	Clathrin-mediated endocytosis
	Cytochalasin-D	5 $\mu g \cdot mL^{-1}$	Disrupt actin filaments and Macropinocytosis
	Filipin	1 $\mu g \cdot mL^{-1}$	Caveolae-mediated endocytosis
**Energy-dependence**	Sodium azide	1 $\mu g \cdot mL^{-1}$	Active transport inhibitor
	4ºC	-	Passive diffusion and active transport inhibitor
